# The cost of saying no: general practitioners’ gatekeeping role in sickness absence certification

**DOI:** 10.1186/s12889-024-17993-1

**Published:** 2024-02-12

**Authors:** Eivor Hovde Hoff, Kristian B Kraft, Cathrine F Moe, Magne Nylenna, Kristian A Østby, Arnstein Mykletun

**Affiliations:** 1 Norwegian Institute of Public Health (NIPH), Cluster for Health Services Research, Postboks 222, Skøyen, Oslo, N-0213 Norway; 2 Office of the Auditor General of Norway, Oslo, Norway; 3 Department of Community Medicine, UiT – The Arctic University of Norway, Tromsø, Norway; 4 Institute of Health and Society, University of Oslo, Oslo, Norway; 5 Centre for Work and Mental Health, Nordland Hospital Trust, Bodø, Norway; 6 Faculty of Nursing and Health Sciences, Nord University, Bodø, Norway; 7 Centre for Research and Education in Forensic Psychiatry and Psychology, Haukeland University Hospital, Bergen, Norway; 8 Myrens verksted 3L, Oslo, 0476 Norway

**Keywords:** General practitioners, Gatekeeping, Sickness absence

## Abstract

**Background:**

General practitioners (GPs) have an important gatekeeping role in the Norwegian sickness insurance system. This role includes limiting access to paid sick leave when this is not justified according to sick leave criteria. 85% of GPs in Norway operate within a fee-for-service system that incentivises short consultations and high service provision. In this qualitative study, we explore how GPs practise the gatekeeping role in sickness absence certification.

**Methods:**

Qualitative data was collected through six focus group interviews with 33 GPs, working in practices with a minimum of four practising GPs, in different geographical regions across Norway, including both urban and rural areas. Data was analysed using Braune and Clarke’s thematic analysis approach.

**Results:**

Our results indicate that GPs’ sick-listing decisions are largely driven by patient demand and preferences for sick leave. GPs reported that they rarely overrule patient requests for sickness absence, including in cases where such requests conflict with the GPs’ opinion of whether sick leave is justified or benefits the patient. The degree of effort made to limit unjustified or non-beneficial sick leave seems to depend on the GPs’ available time and perceived risk of conflict with the patient. GPs generally expressed dissatisfaction with their role as certifiers of sickness absence.

**Conclusion:**

Our study suggests that GPs’ decisions about sickness certification is largely driven by patient preferences. The GPs’ gatekeeping function is limited to negotiations about grade and duration of absence spells.

**Supplementary Information:**

The online version contains supplementary material available at 10.1186/s12889-024-17993-1.

## Background

Statutory paid sick leave systems are common across the OECD and protect employees’ income in the form of sick pay or sickness benefits [1]. In most OECD countries, gatekeeping systems are implemented to reduce costs, ensure appropriate care, and prevent excessive or unwarranted use of social security benefits [2]. General practitioners (GPs) are typically entrusted with the gatekeeper role and certify sickness absence spells exceeding a certain duration.

During the last two decades, increasing rates of long-term sickness absence and disability insurance rolls have raised important policy concerns in many OECD countries [1]. High levels of long-term sickness absence and increasing numbers of disability benefit recipients not only entail significant public expenditure but also carry implications for individuals’ health and socioeconomic well-being. Long-term sickness absence is associated with a high risk of never returning to work [3] and reduces individuals’ future earnings and employment prospects [4]. In recent years, increased attention has also been put towards the potential negative consequences inactivity (full-term absence) can have on individuals’ healing, especially for individuals dealing with mental health issues [5].

In the OECD, Norway has the highest relative number of sickness absence days and disability recipients [6]. On any given day, around 7% of the workforce is absent from work, compared to the OECD average of 3%. Public and mandatory private spending on disability and sickness benefits constitutes around 5% of GDP in Norway, compared to the 2% OECD average [6]. The majority of long-term absence spells are due to musculoskeletal diseases and mental illness, disorders that also account for the majority of disability benefit awards [7]. Despite policy reforms introduced in 2004 and 2010 aimed at reducing sickness absence levels through different activity requirements [8], sickness absence and disability insurance rates remain high [9, 10]. A recent study concludes that Norway has been unsuccessful in curbing sickness absence levels, compared to developments in Sweden and the Netherlands [10].

The OECD has pointed to the Norwegian sickness insurance scheme’s lack of financial incentives for both employees and employers [6]. Norwegian employees are entitled to full wage compensation during sickness absence for up to 12 months. Combined with strong protection against dismissal due to sickness within the first year, might incentivise employees to be absent longer than necessary [11]. Employers cover the first 16 days of absence, giving employers a financial incentive to reduce short-term spells. Despite certain activity requirements, employers however lack (financial) incentives to curtail longer absence spells [6], and might even have a disincentive to reintegrate long-term absent employees [12].

The curbing of unwarranted or non-beneficial sickness absence in Norway is thus highly reliant on GPs’ gatekeeping practices. GPs certify most absence spells exceeding the self-certification period (3–16 days, depending on workplace agreements) [13], and certify disability insurance applications. Sickness absence certificates are granted based on assessments of the medical grounds for a lack of or reduced ability to work. Based on the notion that inactivity (full-term absence) is not considered beneficial for many patients, graded (partial) sickness absence is recommended as the default choice in any case possible [14]. Employees are generally not entitled to sick pay due to normal responses to adverse life events (illness, death of others, funeral, workplace conflicts). Nonetheless, such events can potentially lead to symptoms or perhaps even illness, either acutely or over an extended period.

One rationale behind gatekeeping is that GPs, with their medical expertise, are considered better informed than patients as regards necessity of treatment, taking into account both the patient’s and society’s interests [15, 16]. As certifiers of sickness absence, GPs are protectors of the public purse from unjustified use of welfare benefits. In sickness certification tasks, gatekeeping comes into play when a patient’s preference for sick leave conflicts with either the rule-based justification for a sickness certificate or the GP’s assessment of the patient’s best interest in terms of treatment and healing.

Reibling & Wendt (2012) have pointed out that effective gatekeeping relies on the existence of incentives for gatekeepers to restrict access to services in necessary cases [17]. 85% of Norwegian GPs earn their salary through public reimbursement from *fee-for-service* (activity, 70%) and *capitation* (patient list size, 30%). Fee-for-service models have been shown to incentivise high service provision, for example by reducing consultation lengths and increase the number of visits [18–20], while capitation incentivises keeping current patients on the list.

Studies show substantial variation in GPs’ gatekeeping strictness, affecting both sickness absence incidence and duration [11, 21–24]. Qualitative studies have shown that decisions about sickness certifications are particularly challenged when patients have subjective (unobservable) symptoms [25–29]. Studies also point to fear of conflict and damage to the doctor-patient relationship [27, 30, 31], patient’s ability to evoke empathy [30], physicians’ communication skills [32], and competition for patients [33] as factors contributing to GPs’ decision-making.

In this paper, we explore how general practitioners practise the gatekeeping role in sickness absence certification.

## Methods

Recruitment of GPs was done by contacting 50 medical practices with a minimum of four physicians via phone and email. The threshold of at least four GPs was set to ensure enough participants for the focus group interviews. To try and achieve a diverse representation of participants, the chosen practices spanned different geographical locations across the country, including both urban and rural areas. Most practices did not respond to our invitation, but we managed to arrange interviews with six medical practices, with a total of 33 GPs. The median age of participants was 42, 20 participants were female (61%) and 25 were specialists (76%). In four medical practices, all GPs were self-employed, remunerated via fee-for-service and capitation. In one practice, all but one participant followed this model. In the final practice, all participants were employed by the municipality with fixed salaries. Since interviews were conducted during work hours, GPs were compensated NOK 2000 for participation. Data was collected from September to December in the year 2022.

Interviews were conducted using a semi-structured interview guide. The interview guide was developed based on previous research’s findings and our knowledge about the field. After the first interview, the interview guide underwent a minor revision. During data collection, the prioritising of some questions was changed upon saturation of certain topics. The topics discussed included (i) when and by whom sickness certification is suggested, (ii) information needed to assess whether the conditions for sickness certification are met, (iii) rejection of sickness certification requests, (iv) sickness absence certification in cases where the patient has unobservable symptoms (in particular anxiety, depression, fatigue, pain), and (v) time spent on sickness certification tasks (see supplementary file 1). Data collection for this study was conducted concurrently with another study on GPs’ reflections on the fee-for-service system, allowing for efficient gathering of data for both studies.

Both the first and second author participated in the data collection and alternated having the role of moderator, except for one interview where the first author conducted it alone. All interviews were recorded using an audio recording device. Recordings were transcribed verbatim, and coding of the material was done in NVivo.

Transcripts were analysed using the thematic analysis approach, following Braun and Clarke [34]. The coding of the data material was done using an inductive approach, allowing for the identification of themes and patterns directly from the material. Themes were developed and refined through a recursive process, which involved a continuous revision of the data material against codes and themes. When defining themes, weight has been given to the prevalence of views and statements across participants and groups, as well as statements describing phenomena central to the research question. Contrasting opinions and views are presented when participants’ responses varied notably.

## Results

“*We all know what is the optimal way to do it, who shouldn’t get a sick note and how close the follow-up should be, but why don’t we do it? You mention time pressure, so I think sometimes with sick notes, in the short term, it solves a problem there and then, and the patient is satisfied. Conflict, it drains so much energy, it affects the relationship and the trust, so sometimes I think… I think the times I have done something on a poor basis, it has been to preserve the patient’s trust, or because it was an easy solution. Sometimes because I didn’t know better maybe.*” - Male GP (#28).

Through the analysis we identified four major themes in the discussions about sickness certifications: (i) patient demand and preference for sick leave, (ii) gatekeeping practices in sickness certification tasks, (iii) conditions limiting gatekeeping, and (iv) perspectives on the gatekeeping role and sickness insurance system.

### Patient demand and preference for sick leave

Participants stated that sickness certification is more often requested by patients rather than suggested by the GP. Participants described suggesting sick leave as a treatment for patients’ symptoms as an exception to the rule of patients directly or indirectly asking for such leave.

“*It’s very rare that we are the ones suggesting, like, now I think you should be issued a sick note. It’s very rare, so that one can remember those times, I think.*” - Female GP, specialist (#24).

Exceptions included patient cases where the GP assessed the patient to be in need of taking a break, to prevent exhaustion or burn-out. In these cases, the patients might have a strong preference against being issued a sick note, in which case GP would convince the patient to accept to be sick-listed.

“*I have also become more focused on limiting patients often, especially if it is a burnout issue. They often think they will be back again after two weeks, then I say that no, I don’t think that happens, and just be completely honest about it quite early, that no, you have a long, you actually have a long way to go.*” - Male GP, specialist (#17).

Throughout the discussions, participants also described how they felt that some patients would expect being issued a sick note, regardless of the GP’s assessment of the justification or benefit of sick leave. Examples given included encounters with patients expressing “having the right” to a sick note, having “certified themselves” or arrived with “service orders” for certified absence, irrespective of their health condition or work capacity.

“*It happens quite often that the patient doesn’t suggest anything at all, but they say they’ve already signed themselves off sick. That it’s already done, they’ve managed it all by themselves. They just need a paper confirming it.*” - Male GP, specialist (#12).

Participants frequently referenced the language commonly used in society about certified sickness absence, such as “I have to sick-list myself”. Participants expressed being at the receiving end of a “sickness certification culture” where a “low threshold” for requesting sick leave exists. Many referred to cases from the news where politicians had “taken sick leave” due to causes that appeared to not be health related, and thought that this influenced this culture.

“*If you read the newspapers and listen to politicians and so on, there’s that statement, ‘I had to sign myself off sick’, as if one does it by oneself as if it’s not a [medical] assessment.*” - Female GP (#20).

### Gatekeeping practices in sickness certification

Participants consistently distinguished between patient cases with objective medical symptoms, such as respiratory diseases or a broken leg, versus those with subjective symptoms like anxiety, depression, fatigue, pain and nausea. When the patient had clear medical symptoms, decisions were deemed straightforward, allowing the GPs to rely on their medical expertise to determine the necessity and duration of absence. In contrast, cases reliant on patients’ self-reported symptoms posed challenges and represented the majority of sick leave requests. When discussing sickness certification tasks and challenges, participants most often referred to the latter cases.

#### Rejection of sick leave requests

When asked about whether and when rejection of patient requests for sick leave happens, participants consistently reported that they rarely reject such requests.

“*Rarely. It is rare. One does try to, can have a discussion about it of course, but if there are any patients who are absolutely, completely clear that they are not going to be able to go to work because it is so difficult, then it doesn’t happen so often in my office at least, that I refuse.*” - Female GP, specialist (#7).

Many participants expressed a general reluctance towards declining patients’ requests for sick leave. Participants held the view that overruling requests would either not be accepted by the patient, not benefit the patient in terms of their well-being, or not alter the final outcome (sickness absence).

“… *there are some* [patients] *where it is difficult to understand why they are unable to work with minor problems. My experience with trying to pressure them to work anyway is relatively poor. Because they can’t do it, and it ruins the relationship with the patient, and they end up being signed off sick eventually anyway, just with something else.*” - Male GP, specialist (#19).

Examples of rejected requests were few, limited to cases the participants deemed non-judicious, i.e. clearly outside the eligibility criteria for sick pay. This could be when the reason for the request was other people’s illness, lack of prioritisation of personal tasks, or work conflicts. Nonetheless, responses to these types of requests still seemed to vary among participants. The quotes below illustrate different responses to patients requesting sick leave due to work conflict.

“*If there’s a conflict at the workplace, sometimes it might be appropriate to say that no, this isn’t… there needs to be a dialogue between you and your employer, and it’s not something to be signed off sick for just because you can’t go to work due to disagreements.*” - Female GP, specialist (#7).

“*Yes, often* [work] *conflicts. In such cases, it’s a bit more challenging to achieve that agreement. It’s usually wise to initiate sick leave while simultaneously starting the dialogue with occupational health services, the manager, and arranging a dialogue meeting and all that. Occasionally, things resolve themselves, and many times, in my experience, the best solution for the patient is to just find another job and move on from the current one*.” - Male GP, specialist (#16).

In the case of other people’s illness or adverse life events, participants expressed more difficulties in rejecting or contesting requests, often due to the patient’s situation evoking empathy from the GP. In these cases, some participants were more adamant to refuse the request, while others scrutinised further for symptoms that could warrant a certified absence.

“*If there’s a violation of the rules, so to speak, if someone wants a sick note due to reasons other than their own illness, I have to explain that it’s not possible. But sometimes there’s an accumulation of various unfortunate events which result in patients genuinely… call it a psychological reaction, a stress reaction* [that causes them to] *not be able to work. But if it’s clearly not because of their own illness, then we have to say no.*” - Male GP, specialist (#4).

“*In these types of situations* [death in the family], *I feel like I almost have to put words in their mouth… They do have a lot of symptoms, but you kind of feel like you have to search a bit, ok, do I have any symptoms here, signs of their own illness, then it can be noted as whatever it is, psychological reaction, stress-induced.*” - Male GP (#28).

#### Negotiate grade and duration of sick leave

A common approach when responding to patient requests for sick leave was to engage in “negotiations” with the patient about the grade or duration of the absence spell, to reach a solution that both parties could accept.

“*Often, I try to negotiate a graded one, rather short days. They come in expecting two weeks, so I try like, ‘three days?‘. If they want a week at 100%, I say 50% to try and negotiate.*” - Female GP (#2).

“*Often, it’s a bit like you have to ‘roll with the punches’, so to speak, that you have to try to make a suggestion and then, no, that doesn’t work, no, ok, but maybe we can try something else, and give and take a bit then. Sometimes you have to certify full-term for a period, and then see, ok, but we can certify full-term for two weeks and then come back and look at the plan, that you should prepare yourself a bit to maybe start working a bit.*” - Female GP, specialist (#33).

Participants differed in their views on the benefits of graded sick leave. Some actively negotiated a graded sick leave as opposed to full-term leave in almost all cases. Others believed that graded sick leave could result in an unnecessary long-term absence, referring to experiences with absence spells being “dragged out”.

“*Where I see we have a significant role is* […] *engaging in a dialogue with patients about the degree of sick leave. Most patients often come with the impression that they can either work or they can’t, and this applies both when they start the sick leave and also when they are ending it. And the only thing we know that limits sickness absence levels in Norway is graded sick leave; it’s the only thing that science has proven to reduce sickness absence levels.*” - Male GP, specialist (#21).

“*I increasingly feel that* [the gradual return process] *often contributes to prolonging the situation. If I think that in this case it’s better to reach the finish line instead of jumping in too early, only to end up having to start over, then I think, yea, that’s what I believe.*” - Male GP, specialist (#14).

During negotiations with the patient, participants reported to also try and educate the patient about the social and health related benefits of maintaining their routine and being present at work, regardless of the grade or duration of the sick leave. This included recommending different types of physical or social activities, including maintaining contact with their employer and colleagues. Others reported using language such as “timeout” to signify the expected duration of the absence spell or inform the patient that “at the end of this absence spell, you will not be ill anymore”.

#### Limiting unnecessary long-term or non-beneficial absence spells

Participants shared the view that full-term absence from work over longer periods might be harmful, in particular to patients with mild mental health problems, medically unexplained physical symptoms, or drug addictions. Participants described how patients in these cases might experience either no improvement or worsened symptoms from being on sick leave.

“*Yes, I sometimes think some get worse from being on sick leave.*” - Female GP, specialist (#7).

“*If they don’t come back, the longer time it takes and…*” - Male GP, specialist (#6).

“*Yes, it can be harmful.* […] *With mild mental health issues, I often think that they don’t get better from being on sick leave. But they have to be on sick leave because they can’t handle the job* […] *so they need the sick note anyway, even if both me and perhaps the patient know that it’s not the best.*” - Female GP, specialist (#7).

When discussing the use of sick leave to treat patients with symptoms like anxiety, depression, fatigue, and pain, many participants stressed that the effectiveness of sick leave varies significantly based on individual cases. They highlighted the difference between acute stress or burnout, long-term fatigue or depression, and anxiety problems. In acute cases, many agreed that full, short-term sick leave could be effective in aiding patients to better manage their lives. In the other cases, several participants advocated the use of partial rather than full-term leave and emphasised being aware of the risks of aggravated symptoms and/or not returning to work.

“*If someone has stretched the limit too far and would benefit greatly from two weeks, then that’s completely unproblematic, giving them a little restart. But yes, if there is clear depression, I mean, you feel it and you know that there have been several sick leaves already, then that is different than if it is a kind of one-time occurrence.*” - Female GP (#22).

“*A large group we often have on follow-up*, [are] *those with medically unexplained symptoms. And there I think, initially, they are poor candidates for full sick leave, there can be a lot of aggravation* [of symptoms] *there. I believe making them more passive, that’s not good for them. In those situations where it sometimes, if it results in [sick leave], I try to make it as short as possible.*” - Male GP (#28).

Some participants pointed out that determining the underlying health issue and assessing the potential benefits of sick leave, is not always straightforward in these cases.

“*I don’t think there’s a clear divide. There are some where it’s depression, and then there are some where it’s overload and tiredness. And then there’s this divide in between where it can flow from one state to the other, and that divide can be difficult when evaluating sick leave. Where I have used the most effort is when I conflict with myself, when I become unsure if my assessments are correct. When I think the patient should not be on sick leave or should not be sick to that degree, but the patient insists that they should, that they need it. When I either say no and get an angry patient, or say yes and go home feeling I have compromised my professionalism.*” - Female GP, specialist (#18).

As a strategy to ensure that patients return to work, many participants advocated making a plan for the duration of the absence spell and/or agreeing on a gradual reduction of absence percentages over time. Participants seemed to have somewhat different strategies when it came to the concreteness of the plan and how they arranged the patient follow-up. The quotes below illustrate a defined plan from start to finish, whilst the other a more wait-and-see approach.

“*I usually say that there are many numbers between zero and a hundred*, […] *and then I listen a bit to what they think, and then we find some number that fits, and then I also write a follow-up at some point, hear how it goes and…*” - Female GP, specialist (#27).

“*It’s essential to provide clear guidance during patient follow-ups.* […] *By laying out expectations, patients have a clearer understanding of what lies ahead. You can even pre-arrange the sick leave, setting two weeks at 50%, followed by two weeks at 40%, and then another two weeks at 30%. I find this approach works very well; it unfolds seamlessly*.” - Female GP (#20).

Several participants shared experiences with patients ending up in long-term absence spells, observing how the threshold of returning to work would increase the longer the duration of the absence. The dialogue below describes how initial short-term leave could lead to longer absences and an increasing reluctance or inability of patients to return to their regular routines or work.

“*We don’t get them back.*” (#14).

“*Yes… Well, you’re right about that. It usually starts with a two-week sick note. Then they come back, things haven’t changed, they don’t feel any better, and after a dialogue, it usually extends to a longer sick note. I’m not sure if it becomes a crutch for the patient or what happens, but something does.*..” (#16).

“*Yes, and then there tends to be some sort of aversion that develops. If you’ve been away for a while, the threshold to return keeps rising.* […] *I can’t predict when it’s going to happen. But I’ve experienced it a few times where I think, yeah, that wasn’t so smart.*” (#14).

Dialogue between participant #14 (Male GP, specialist) and participant #16 (Male GP, specialist).

A few participants described how they, based on similar experiences as those mentioned above, had changed their strategy for patient follow-up after observing patients not feeling better after being on sick leave.

“*There are certain strategies and adjustments I’ve adopted over time to potentially reduce the duration of absence spells. Previously, I would schedule a follow-up appointment right before the end of a patient’s sick leave to ensure they were fit to return to work. However, this often resulted in an extension of the sick leave, as patients felt they weren’t ready. Now, I’ve shifted to outlining a comprehensive sick leave plan during the initial meeting* […], *and schedule a follow-up a few weeks* after *the sick leave has concluded. This gives the responsibility to the patient to reach out if things aren’t going as planned. I’ve observed that this approach tends to decrease the duration. Excessive monitoring can sometimes inadvertently lead to prolonged sick leaves.* […] *The barrier to proactively extending sick leave becomes much higher than simply attending a pre-arranged appointment and getting an extension.*” - Male GP, specialist (#21).

Regarding patients that were enrolled in work allowance assessments, post 12 months of sickness absence, some participants described how the contact with these patients diminished.

“*You can often see it with patients who have been sick for a year and then transition to work assessment allowance (AAP)*.” (#8).

“*How rarely they come in?*“(#12).

“*Yes, then you don’t see them for half a year.*” (#8).

“*No, haha… And you wonder where in the world they went.*” (#12).

Dialogue between #8 (Male GP, specialist) and #12 (Male GP, specialist).

Two participants provided examples where they successfully had prevented patients from becoming dependent on disability benefits.

“*She was on the verge of transitioning to disability, and felt it was unfair as she saw many others were on sick leave with fewer complaints than hers, while I was pressing her to return to work. She felt somewhat invalidated and not fully cared for. Eventually, she did return to work and has been working full-time since. Reflecting on her case, had she continued on to disability benefits, her life trajectory might have been very different. Prolonged sick leave can impact individuals’ perceptions of their health, functional capacity, and ability to work if they aren’t given appropriate guidance on managing their sick leave decisions.*” - Female GP, specialist (#18).

*“These are some of those few uplifting stories that we live for when we take those fights with the patients. Then, for every success story, there are maybe 10 or 15 or more that don’t amount to anything.”* - Male GP, specialist (#21).

### Conditions limiting gatekeeping

#### Information asymmetry

Participants consistently described how, in many cases where sick leave is requested, the patient has symptoms that are not readily observable by the GP beyond the patient’s self-report. Participants expressed not being able to contest the patient’s symptom descriptions and feelings of being ill or not able to work.

“*It’s not easy to say… how sick the individual is. So if they absolutely do not feel able to work, I find it difficult to argue… Then it’s a bit like ‘yes, but I am sick’, ‘no, you are not sick’. How am I supposed to say no on any level?*” - Male GP, specialist (#6).

This information asymmetry was also evident when discussing assessments of the patients’ work ability. Participants stated that they found it difficult to challenge the patient’s description of not being able to work, especially when they lacked sufficient information about the patient’s employment situation and the patient was persistent about their employer’s lack of ability to facilitate.

“*So, we are a bit at the mercy of the answers we get then, we can’t call every employer and ask if it’s true that they can’t accommodate, for example. It’s not so rare that people say no.*” - Female GP, specialist (#24).

“*There are quite a few who say that it’s either 100% sick leave, or 100% work. That they can’t do any other tasks, and that the employer can’t make accommodations, period.*” - Female GP, specialist (#25).

“*So, you become a bit of a hostage.*” - Female GP, specialist (#24).

#### Risk of conflict with patient and damage to the doctor-patient relationship

Drawing from experience, participants described how contesting patient requests could result in uncomfortable confrontations or conflict. Such conflicts or disagreements led to both the GP and patient being dissatisfied, and several participants admitted that “*sometimes it’s easier to write a short sick note than to kind of […] argue*”. Some participants also described how the workday would end up feeling unbearable if conflicts with patients occurred regularly.

“*I sort of want my everyday life to be pleasant. I’m always trying, like, to meet, if there’s a basis for it, I’ll accommodate the sick note, but sometimes I get so tired if I’m supposed to start arguing with the patient all the time. If you have 2–3 of those in a day, you end up pretty worn out by the end of the day.… I go along with it because I can’t bear it; I get mentally exhausted if I have to fight with the patient every time.*” - Female GP (#2).

Participants emphasised how conflict ultimately could damage the doctor-patient relationship, since many patients would end up feeling mistrusted or not taken care of by the GP.

“*So you might break the good relationship that has been built up over many years when there’s trust in the doctor and a good collaboration with the patients, then the sick note gets in the way.*” - Female GP, specialist (#32).

#### Time constraints

*“Well, often the easiest thing for us is to write a 100% sick leave for four weeks, see ya. There’s no doubt about that.”* - Male GP (#31).

*“That’s what gives us the least amount of work. But, we don’t do that very often.”* - Female GP, specialist (#33).

*“No, of course not.”* - Male GP (#31).

*“We understand that it’s not wise, so we spend time on it, time we don’t really have.”* - Female GP, specialist (#33).

When discussing time constraints and use of time on sick leave consultations, participants described how understanding the patient’s underlying problem, discussing the benefits of sick leave, contesting patients’ preferences, and follow-up of patients as the most time-consuming tasks.

“*You have 20 minutes, right. The patient has to come into the office, you have to be presented with the issues, you might have to examine, make a plan, document, write a sick note, all in 20 minutes, right. And this probably leads to* [more] *sick notes and longer spells* [than it should be], *because you simply need time to do all that. Take dizziness. There are lots of things you need to get done and examined, and then it also depends on getting all the information* [from the patient], *and that’s difficult in maybe 10 minutes of effective conversation, it takes a bit of time for that to come out. And then maybe there has been an assault that is the cause of everything, right.*” - Male GP (#31).

Contesting patient claims or discussing the benefits of sickness absence were described as the most time-consuming. Some expressed a certain resignation as regards their ability to convince patients that sickness absence was not warranted or beneficial.

*“I used to reject sick notes much more in the past. I questioned them much more when patients came and asked for a sick note. I realised that I’m not getting anywhere; I spend five times as long on that consultation, without gaining much, most of the time. There might be a few instances where we come to a mutual agreement. But when I experience that immense pressure in daily life, I feel that’s a battle I just don’t have the energy to fight.”* - Female GP, specialist (#10).

Some participants acknowledged that time constraints could lead to insufficient monitoring of patients on sick leave, potentially resulting in extended absences that are not beneficial in the long term.

“*I also think that much of the blame can lie in both that we are pressed for time, so I don’t have time to follow them up the way I perhaps would have done to quickly reverse a difficult trend. And then there are long waiting lists. If there are mild to moderate issues, right, you think maybe they could get quickly back to work if they had a therapeutic conversation alternative that addressed that interest, but they don’t. In these cases I think sick leave becomes harmful because they are kind of in a waiting zone, where they are too sick to be in a 100% job and they actually feel they get worse because they are more isolated and they have fewer of these other safety measures, and I unfortunately am a bit too busy at work to be able to take them in and adequately follow them up.*” - Female GP, specialist (#9).

### Perspectives on the gatekeeping role and sickness insurance system

Several participants conveyed mixed feelings or dissatisfaction with their role as certifiers of sick leave. The dissatisfaction seemed to be primarily related to the GPs’ lack of knowledge about workplace facilitation options, combined with potential conflict risks associated with contesting requests for sickness absence.

“*I really think that the sick leave system has become a bit of a pain to manage, because, as one says, no matter how you twist and turn it, I can describe function from here to kingdom come and hell, but it doesn’t change the fact that the patient goes to work and then the manager says, ‘I don’t want you here because you’re not 100%, you’re not functioning as you should,’ and then the patient is left standing there. And then there’s a conflict between the two of us that really doesn’t need to be there*.” - Female GP, specialist (#9).

Many participants held the view that sickness absence is primarily a case between employers and employees.

“*I kind of think that sick leave is more, I’m not saying that we shouldn’t issue sick notes, but I believe that sick leave is primarily a matter between the employee and employer. Then I’ve been set to be, say, the scapegoat. If I were to have it my way, I would want the sick leave to be a matter between the employer and the worker, and that I was more of a consultant who could have an opinion about the burden of symptoms for the patient*.” - Female GP, specialist (#27).

In many sessions, the participants pointed to the employers’ role and responsibilities in facilitation and reintegrating employees on sick leave.

*“I think that the employer could have been more involved many times. I believe that much of the problem is the reintegration and discussion of function, the possibility for work adjustments.”* - Male GP, specialist (#16).

*“Adjustments, yes.” -* Female GP, specialist (#15).

*“That much more should happen there than in my doctor’s office, I must say. A lot can happen without us doctors being present.”* - Male GP, specialist (#16).

When asked about whether it was the GP or the patient that had the decisive power regarding whether to certify sick leave, participants differed in their views. Some held the view that they were “at the end of the day” the ones with the decisive power, others that the decision was a compromise between patient and doctor, and others again described that in practice is the patient who decides whether or not to be sick-listed.

“*I believe if you asked the patients, they’d say it’s the doctor’s decision. They probably prefer someone else determining their sick leave status. But they come in with an opinion on needing sick leave, so, often, we just follow their lead. We don’t really have the means to thoroughly investigate and challenge their claims.*” - Female GP, specialist (#33).

The conversation below illustrates the varying perceptions of control and responsibility doctors feel regarding their decisive role in sick-listing decisions, highlighting the tension between perceived authority, collaborative patient care, and professional integrity.

*“At the end of the day, we are the ones deciding”* - Female GP, specialist (#7).

*“I often believe, at least for me, it’s an illusion that I control everything entirely… It feels more like a mutual discussion where both parties come to an agreement. I don’t feel like I decide it all by myself.”* - Male GP, specialist (#8).

*“Maybe it’s just an illusion, thinking we have control. But I often feel extremely uncomfortable if I issue a sick note that I can’t fully stand behind. That discomfort is immeasurable to me. If I felt I didn’t have control over it, I wouldn’t be comfortable being a doctor.”* - Female GP, specialist (#9).

## Discussion

GPs reported that decisions about sickness certification are largely driven by patient demand and preferences. Saying no to patient requests for sickness absence entails both social and financial costs for GPs, and challenges their role as gatekeepers for sickness absence. The GPs describe how challenging requests for sick leave may lead to unpleasant conflicts with the patient, with possible harms to the doctor-patient relationship. There is a notable information asymmetry in many of these consultations, where GPs feel they have to rely on the patient’s descriptions of symptoms and possibilities for workplace adjustments. This is particularly difficult when the patient’s symptoms are not readily observable by the GP beyond the patient’s self-report. As a result, decisions about sick leave are to a large degree based on the patient’s preference, even when it conflicts with the GP’s own medical judgement. Gatekeeping efforts in sickness absence decisions are described as in many cases being unpleasant and stressful.

This study is in line with previous qualitative studies on GPs’ gatekeeping decisions in sickness absence certifications, in particular the limited gatekeeping due to information asymmetry and risk of conflict with patients [25, 26, 29, 30, 33, 35]. For several of these studies, data was collected 15–20 years ago [26, 29, 33]. 20 years on, our findings are largely the same, suggesting that many of the same mechanisms challenging GPs’ gatekeeping of sickness absence are equally relevant today. Our study contributes with the mechanism of time constraints contributing to the GPs’ ability and willingness to limit unjustified or non-beneficial sickness absence.

In the current Norwegian fee-for-service model, GPs will generally benefit financially from shorter consultations. The GPs state that contesting patient requests for certified sickness absence is substantially more time-consuming than granting the request. Contesting sickness absence requests involves negotiating duration and grading, which requires specific understanding of not only the patient’s health situation but also how it affects their work tasks and the potential for workplace adjustments. Studies find that fee-for-service GPs have shorter consultation times compared to their fixed salary counterparts [36]. As fee-for-service incentivises shorter consultations, it may be that it also contributes to increased sickness absence. This is supported by a study showing that GPs issue sickness certificates more often when paid fee-for-service compared to when they are paid by fixed salary [37].

The gatekeeping system rests on the idea that GPs are better equipped than patients to evaluate and determine what is in the patient’s and society’s long-term interest. Most long-term absence spells are certified for musculoskeletal disorders and mild or moderate mental disorders [38], disorders that are difficult to verify [35]. For some of these diagnoses, the GPs in our study consider sick leave to be potentially harmful. It may lead to inactivity, isolation, loss of daily routines, and loss of social interactions at work. The GPs report being aware of these risks and making efforts to promote partial sickness absence as an alternative to full absence, or try to reduce the duration of absence spells. Despite being aware of these risks, the gatekeeper role is often compromised, and the patient’s preferences for sickness absence outweigh the GP’s medical opinion about the patient’s long-term interest.

Non-medical factors related to the workplace or private life, may also sometimes contribute to a patient’s request for sick leave. Examples include workplace conflicts and family related problems. In general, such issues are not considered adequate causes for sick leave certification. Some GPs still certify absence in such cases, acting as an advocate for the patient’s preference rather than a gatekeeping bureaucrat. In these cases, GPs report to enquire about common symptoms like tiredness or sleep problems to justify the certification of absence.

Another important purpose of the gatekeeper role is the safeguarding public funds and ensuring the judicious use of welfare services. On behalf of society, the GP is mandated to be the guardian of the public purse. Among OECD countries, Norway has the highest level of sickness absence [6]. The OECD attributes this to the absence of financial incentives for both employers and employees for sickness absence exceeding the employer period, after which all costs are covered through public funding. As a result, the GPs’ gatekeeping role is the only remaining mechanism to avoid misuse of the sickness absence system. Our results consistently suggest that current gatekeeping of sickness absence is, at best, tenuous.

The combination of lack of gatekeeping and financial incentives is likely to contribute to Norway’s high level of sickness absence. GPs express dissatisfaction with their gatekeeping duties in sickness absence certification, with many favouring financial incentives for employees or employers to regulate excessive absence. The current remuneration system for GPs, with incentives for short consultations and keeping their patients satisfied, might function as a disincentive for the gatekeeping of sickness absence.

### Strengths and limitations

This study is based on the accounts of 33 general practitioners. The study’s strength lies in examining GPs’ perspectives and approaches in sickness certification, offering valuable insights into their decision-making and their role as gatekeepers. The methodology is limited to the GPs’ self-reported experiences and evaluations, without direct observation of their actual behaviour. Despite potential social desirability bias in such self-reports, participants appeared to candidly express their attitudes and experiences. Nonetheless, we cannot preclude the possibility that some opinions or experiences were withheld by certain participants. One limitation of focus group studies is the possibility of group conformity or dominant individuals overshadowing others. We observed that some participants were less vocal, which could have limited diverging opinions to come forth. However, group discussions also brought out diverse opinions as a result of dialogue between participants. We observed consistent patterns in responses on several themes across the groups.

Participants, mainly practice leaders or their equivalents, voluntarily joined the study. Although this self-selection might suggest a bias, the involvement of other GPs from the same practices, might have mitigated this to some extent. At the same time, GPs from the same practice may have similar perspectives.

## Conclusion

Our study suggests that GPs’ decisions about sickness certification is largely driven by patient preferences. The GPs’ gatekeeping function is limited to negotiations about grade and duration of absence spells.

### Electronic supplementary material

Below is the link to the electronic supplementary material.

Supplementary Material 1

## Data Availability

The dataset generated and analysed during the current study is not publicly available due to participant privacy but is available from the corresponding author on reasonable request.

## Abbreviations

GP: General practitioner
OECD: Organisation for Economic Co-operation and Development
