# Supplementary material for: The cost of saying no: general practitioners’ gatekeeping role in sickness absence certification
Source: BMC Public Health. 2024 Feb 12;24:439. doi: 10.1186/s12889-024-17993-1 (PMC10860288; doi:10.1186/s12889-024-17993-1)
Supplement: Supplementary file 1 — Supplementary Material 1 [file 12889_2024_17993_MOESM1_ESM.docx]

Supplementary file 1: Interview guide

Q1: Both the patient and the general practitioner can suggest sick leave during the consultation. What happens most often?"

Q2: What do you need to know in order to assess whether the patient needs a sick leave note?

Q3: In which cases do you reject the patients' request for sick leave?

Q4: Sometimes patients ask for sick leave for issues that are difficult for a doctor to objectively observe, such as anxiety, depression, fatigue, pain, etc. What are your thoughts on sick leave as a treatment in such cases?

Q5: In which cases can sick leave be harmful?

Q6: Who is the one deciding whether sickness certificates are granted? You? The patient?

Q7: Do you spend a lot of time on conversation/assessment of the patient in matters of sick leave?
